# Eradicating Suicide at Its Roots: Preclinical Bases and Clinical Evidence of the Efficacy of Ketamine in the Treatment of Suicidal Behaviors

**DOI:** 10.3390/ijms19102888

**Published:** 2018-09-23

**Authors:** Domenico De Berardis, Michele Fornaro, Alessandro Valchera, Marilde Cavuto, Giampaolo Perna, Marco Di Nicola, Gianluca Serafini, Alessandro Carano, Maurizio Pompili, Federica Vellante, Laura Orsolini, Annastasia Fiengo, Antonio Ventriglio, Kim Yong-Ku, Giovanni Martinotti, Massimo Di Giannantonio, Carmine Tomasetti

**Affiliations:** 1 National Health Service, Department of Mental Health, Psychiatric Service of Diagnosis and Treatment, “G. Mazzini” Hospital, p.zza Italia 1, 64100 Teramo, Italy; 2 Department of Neuroscience, Imaging and Clinical Science, Chair of Psychiatry, University “G. D’Annunzio”, 66100 Chieti, Italy; federica.vellante@gmail.com (F.V.); giovanni.martinotti@gmail.com (G.M.); digiannantonio@unich.it (M.D.G.); 3 Polyedra Research Group, 64100 Teramo, Italy; dott.fornaro@gmail.com (M.F.); a.valchera@ospedaliere.it (A.V.); laura.orsolini@hotmail.it (L.O.); annastasia.fiengo@gmail.com (A.F.); carmine.tomasetti@aslteramo.it (C.T.); 4 Department of Neuroscience, Reproductive Science and Odontostomatology, School of Medicine ‘Federico II’ Naples, 80121 Naples, Italy; 5 Villa S. Giuseppe Hospital, Hermanas Hospitalarias, 63100 Ascoli Piceno, Italy; 6 Department of Theory, Analysis and Composition, Music Conservatory “L. Canepa”, 07100 Sassari, Italy; marilde_cavuto@virgilio.it; 7 Hermanas Hospitalarias, FoRiPsi, Department of Clinical Neurosciences, Villa San Benedetto Menni, Albese con Cassano, 22032 Como, Italy; pernagp@gmail.com; 8 Department of Psychiatry and Neuropsychology, University of Maastricht, 6221 Maastricht, The Netherlands; 9 Department of Psychiatry and Behavioral Sciences, Leonard Miller School of Medicine, University of Miami, Coral Gables, FL 33114, USA; 10 Institute of Psychiatry and Psychology, Catholic University of Sacred Heart, 00118 Rome, Italy; marcodinicola.md@gmail.com; 11 Department of Neuroscience, Rehabilitation, Ophthalmology, Genetics, Maternal and Child Health, Section of Psychiatry, University of Genoa, 16132 Genoa, Italy; gianluca.serafini@uniroma1.it; 12 NHS, Department of Mental Health, Psychiatric Service of Diagnosis and Treatment, Hospital “Madonna Del Soccorso”, A.S.U.R. 12, 63074 San Benedetto del Tronto, Italy; alessandro.carano@gmail.com; 13 Department of Neurosciences, Mental Health and Sensory Organs, Suicide Prevention Center, Sant’Andrea Hospital, Sapienza University of Rome, 00118 Rome, Italy; maurizio.pompili@uniroma1.it; 14 Psychopharmacology, Drug Misuse and Novel Psychoactive Substances Research Unit, School of Life and Medical Sciences, College Lane Campus, University of Hertfordshire, Hatfield SG141LZ, UK; 15 NHS, Department of Mental Health ASUR Marche AV5, Mental Health Unit, 63100 Ascoli Piceno, Italy; 16 Department of Clinical and Experimental Medicine, University of Foggia, 71121 Foggia, Italy; a.ventriglio@libero.it; 17 Department of Psychiatry, Korea University College of Medicine, Seoul 08826, Korea; yongku@korea.edu

**Keywords:** mood disorders, dopamine, serotonin, glutamate, postsynaptic density, antipsychotics, antidepressants, NMDA, suicide, ketamine, esketamine

## Abstract

Despite the continuous advancement in neurosciences as well as in the knowledge of human behaviors pathophysiology, currently suicide represents a puzzling challenge. The World Health Organization (WHO) has established that one million people die by suicide every year, with the impressive daily rate of a suicide every 40 s. The weightiest concern about suicidal behavior is how difficult it is for healthcare professionals to predict. However, recent evidence in genomic studies has pointed out the essential role that genetics could play in influencing person’s suicide risk. Combining genomic and clinical risk assessment approaches, some studies have identified a number of biomarkers for suicidal ideation, which are involved in neural connectivity, neural activity, mood, as well as in immune and inflammatory response, such as the mammalian target of rapamycin (mTOR) signaling. This interesting discovery provides the neurobiological bases for the use of drugs that impact these specific signaling pathways in the treatment of suicidality, such as ketamine. Ketamine, an *N*-methyl-d-aspartate glutamate (NMDA) antagonist agent, has recently hit the headlines because of its rapid antidepressant and concurrent anti-suicidal action. Here we review the preclinical and clinical evidence that lay the foundations of the efficacy of ketamine in the treatment of suicidal ideation in mood disorders, thereby also approaching the essential question of the understanding of neurobiological processes of suicide and the potential therapeutics.

## 1. The Roots of Suicide: Current Advances in Neurobiology of Depression and Suicidal Behaviors

### 1.1. A Global Phenomenon

Among 800,000 people commit suicide every year, and even many more people attempt suicide [1]. The World Health Organization has estimated that 78% of suicide attempts occur in low- and middle-income countries, and that in 2015 suicide was the second leading cause of death amongst 15–29-year-old young adults globally [2].

A large body of evidence reports that 90% of suicide victims suffer, or have suffered, from psychiatric disorders [3,4], as well as 50% of people committing suicide has been affected by major affective disorders (e.g., major depressive disorder, bipolar disorder) [5,6]. However, alcohol and drug abuse, schizophrenia, and personality disorders have been often associated to suicide, together with affective disorders [7].

As mentioned, the vast majority of people attempting to their own lives belong to low/middle-income social classes, therefore there is a large amount of data claiming that the ten-year economic crisis would have influenced suicide rates, probably by reducing and impairing cares for mental health patients [8].

### 1.2. The Process of Suicidal Behavior

From an ethological and psychobiological point of view, suicidal behavior can be seen as a complicated process in which genetic, neurobiological, environmental and psychological factors imbricate in order to develop the final outcome [9,10]. However, how these factors interact with each other represents an issue so entangled that it is actually extremely difficult to predict who would commit a complete suicide and when [11,12,13]. Indeed, A recent meta-analysis on suicide risk reported that 95% of high-risk patients (i.e., displaying multiple suicide-related characteristics defining a high propensity to suicidal mortality) usually do not attempt suicide, whereas 50% of low-risk patients do, thus confirming the complexity of suicidal behavior processes [14].

Recent research breaks down suicidal behavior into its chronological parts: the ideation, the planning, the attempt/attempts, and the complete suicide [15]. Each phase may have a different duration and intensity, and, although suicidal attempts cannot chronologically precede ideation, they do not always follow it as expected, since suicidal ideas are not always followed by suicidal attempts. However, even if suicidal ideation should be manifest, it has been demonstrated in several studies that clinical interventions may not be efficacious [16]. A lot of studies have reported high rates of persistence of suicidal ideation in people diagnosed with psychiatric disorders, despite adequate treatments. For example: in first-episode psychosis patients, 33% report moderately stable and 7% moderately increasing persistent suicidal ideation over two years [17]; 6.3% of patients with diagnosed depression may experience high suicidal ideation despite 4 months of treatment [18]; 14% of bipolar disorder patients endure suicidal ideation over 6 months of treatment [19]; and lastly, even after hospitalization due to acute attempts or acute ideation, suicidal thoughts have been demonstrated to persist in adolescent patients even after 12 months following discharge from treatment [20]. Anyway, some studies suggest there are possible predictors of suicide, even if ideation is not completely expressed. For instance, Köhler-Forsberg and colleagues have reported that suicide may be predicted by higher baseline depression scores in bipolar patients with persistent ideation [19]. Furthermore, suicide attempts may be considered a significant risk factor for possible completed suicide [21].

### 1.3. Integrated Theories of Suicide

Modern theories try to converge multiple levels of analysis, such as neurobiological (i.e., dysfunctions in the hypothalamic-pituitary axis, abnormal noradrenergic/serotonergic/dopaminergic/glutamatergic neurotransmissions, neuroinflammatory dysregulation) and sociological hypotheses (i.e., the relationship between the individual personality and social/environmental stressful factors), in order to develop and integrated model aiming at finding a “biosignature” of suicide [22,23,24]. For example, a recent study—based on preclinical evidence of the association between the short allele of the 5-HTT (serotonin transporter) gene-linked polymorphic region and greater susceptibility to stress events [25,26,27]—tested the hypothesis that variants in the 5-HTT gene might influence dysfunctional adaptation to stressful events (gene-by-environment hypothesis) by analyzing the birth cohort study of 1037 people in the Dunedin Multidisciplinary Health and Development Study, thus finding that individuals yielding the short allele of the 5-HTT promoter polymorphism exhibited more depressive symptoms, and more propensity to suicidal behaviors in relation to stressful life events than individuals homozygous for the long allele [28].

Moreover, an extensive family-based genetic study analyzing suicide attempters switched the attention from “classical” serotonergic-related pathways to the involvement of GABA and glutamate neurotransmission in suicidal behaviors. Indeed, Sokolowski et al. found that GRIN2B (a glutamate NMDA receptor subunit gene) and ODC1 (a gene coding for ornithine decarboxylase, a rate-limiting enzyme on the polyamine synthesis pathway) seem to be associated with severe suicide attempts, as well as with serious physical assault in childhood and adolescence, which in turn increase the risk of suicide attempts, thereby configuring a gene-by-environment interaction [29].

Finally, according to a diathesis-stress model, recent evidence points out that early stressful life events may increase the diathesis for suicidal behaviors both by provoking epigenetic and neurological changes (e.g., on serotonin neurotransmission, opioids, oxytocin, and HPA axis) and impacting on neurobiological correlates underlying character traits that have been associated with susceptibility to suicide risk (such as impulsivity, emotional dysregulation, poor attachment, chronic pessimism, impaired cognition) (see [30] for an exhaustive review).

## 2. Cognitive Bases of Suicidal Behaviors: The Role of Glutamate Neurotransmission

### 2.1. Cognitive Dysfunctions in Suicide Attempters

As mentioned above, suicide may be envisioned as a complex process involving multiple interactions amongst biological, psychological, and environmental factors. Recent researchers have begun to place suicidal progression within a framework of cognitive schemas involving basic cognition and individual reality interpretation (i.e., perceived burdensomeness, thwarted belongingness) [30]. Basic cognitive alterations have been studied from a neuropsychological point of view and found to be related to abnormal neurocircuitry connections, especially in prefrontal-striatal brain circuits (ventrolateral orbital cortex, dorsolateral/dorsomedial prefrontal cortex, anterior cingulate gyrus) [31], and probably to involve glutamatergic neurotransmission dysregulation in these areas [32].

Cognitive hypotheses of suicidal behaviors are based on the well-validated theories about acquired capability for suicide. The interpersonal-psychological theory of suicidal behavior, elaborated by Thomas Joiner in 2005 [33], suggests that completed suicides require three specific characteristics: a sense of thwarted belongingness, a sense of burdensomeness, and an acquired capability for suicide, which is characterized as the habituation to fear, pain, and death by means of continued and progressive exposure to painful and provocative events, thereby causing a sort of desensitization. Moreover, the acquired capability for suicide must necessary entangle with reasons to attempt suicide, in order to finalize the progress to completed suicide. Different studies have reported that feelings of loneliness or being unwanted, together with the will for auto-punishment, as well as the necessity of get relief from a terribly distressing state of mind, may represent the more prominent reasons to commit suicide [34].

These theories may be re-examined from a “cognitive” point of view in order to identify the putative cognitive mechanisms at the basis of suicidal behavior, such as the effects of the negative thoughts and overwhelming emotions of affective patients on their perception of environmental stimuli, which could be defined as attentional bias. Indeed, different studies have reported that often psychiatric patients pay much attention to environmental cues that are specifically related to their suffering, and in particular the sense of hopelessness, burdensomeness, and loneliness may cause extreme difficulties in patients to find solutions to their problems other than suicide [35]. Attentional bias may be measured by specific neuropsychological tests exploring executive functions, such as the Stroop paradigm, which requests that patients name the color in which some words are written. A recent study reported that depressed patients may perform defectively on the Stroop test, when asked to name colors of suicide-related words [36]. These results confirm previous findings of worse performances in memory, attention and working-memory tasks by non-violent, depressed patients who have attempted suicide in the past, as compared to depressed non-attempters, and suggest that executive dysfunctions in people who have attempted suicide may be related to specific forms of suicidal behavior [37].

A particular mention should be made of the relationship between suicide and impulsivity, which has been widely investigated. Impulsivity has been associated with suicidal behavior in a large amount of studies [38] and it has been regarded as a heritable trait [39]. Impulsive decision-making has been related to suicide attempts in bipolar patients [40], and depressed patients with higher impulsivity have been reported to show higher rates of suicide attempts regardless of the severity of their depressive symptoms [41]. Moreover, impulsivity and aggression have been both correlated with high-lethality suicide attempts in borderline personality disorder patients, and associated with abnormal volumes across multiple fronto-temporal-limbic regions deputed to cognitive control of emotions and behavioral planning [42].

### 2.2. Glutamate Neurotransmission Dysregulation as the Foundation of Suicidal Cognitive Biases

Cognitive flexibility is a critical executive function that represents the ability to adapt behavior in response to environmental changes. Its dysfunctions have been largely regarded as contributing to the onset of several psychiatric diseases, such as depressive, bipolar, and anxiety disorders [43]. Functional studies have associated the deficits in cognitive flexibility showed by depressed patients to specific alterations in the structure and connection of the prefrontal cortex (PFC) [44,45].

Glutamate neurotransmission dysregulation seems to represent the core neurobiological foundations the abovementioned functional alterations. Indeed, reduced glutamate/glutamine and GABA levels (positively correlated with each other) have been demonstrated in the prefrontal cortex of unmedicated depressed patients using proton magnetic resonance spectroscopy [46]. In order to corroborate these findings, reduced levels of NR2A and NR2B subunits of the *N*-methyl-d-aspartate (NMDA) ionotropic glutamate receptors have also been found in postmortem PFCs of major depression patients, together with a concurrent reduction in PSD-95, a scaffold postsynaptic protein that is essential in NMDA trafficking and transductional pathways, thus suggesting abnormalities in the whole glutamate signaling in brain areas deputed to cognitive tasks [47]. In animal models, acute stress may enhance glutamate neurotransmission in PFC [48], and the increase in glutamate-mediated activation may potentiate working memory performances [49], whereas NMDA blocking impairs cognitive flexibility [50].

Chronic stress, instead, may cause a sustained impairment by means of the suppression of glutamate receptors in PFC, and may consequently provoke its dysfunctionality [51]. A recent preclinical work has demonstrated that chronic stress may resemble the PFC-dependent attention set-shifting deficits induced by NMDA blockade, and that these deficits are associated with an attenuated afferent activation of the PFC as well as a reduced acute stress-induced glutamate efflux in PFC, thus configuring impaired cognitive flexibility responses [52].

Several studies have confirmed the crucial role in suicidal behavior of glutamate neurotransmission dysregulation in cognition-associated brain areas. Indeed, glutamine synthetase-expressing glial cells have been found significantly elevated in the dorsolateral PFC and in orbitofrontal cortex of schizophrenia patients who completed suicide, as compared with controls and non-suicidal patients, thereby suggesting that a disruption in glutamate-glutamine-GABA cycle in these brain areas may have a significant impact on suicidal behavior [53]. In suicidal patients a significant increase in glutamic acid decarboxylase (GAD, the enzyme deputed to GABA synthesis starting from glutamate) neuropil has been reported in the hippocampus [54]. Finally, a selective PFC up-regulation of an alternative full-length transcript of GAD has been recently observed in suicide-completer schizophrenia patients [32].

As mentioned above, an extensive family-based genomic study reported a significant association of GRIN2B (coding for a glutamate NMDA subunit) with severe suicide attempts, as well as a significant gene-by-environment relation with a history of physical assault in childhood and adolescence, increasing suicide risk [29].

Impaired glutamate neurotransmission in the cingulate cortex has been also directly correlated with impulsivity and cognitive dysregulation [55]. A recent study demonstrated that glutamate levels measured in cerebro-spinal fluid may be directly correlated with impulsive aggression in both subjects diagnosed with personality disorders and healthy volunteers, independently from their pathology [48].

Moreover, given that clozapine has been universally recognized as a superior treatment for both suicidality and aggression/impulsivity [56,57,58], the finding that this antipsychotic may potentiate NMDA receptor function by inhibiting Sistem A-mediated glycine transport could account for its differential therapeutic properties as compared to other antipsychotics for suicidal behavior [59].

## 3. Eradicating the Roots of Suicide: Neurobiological Bases of Anti-Suicidal Effects of Ketamine

### 3.1. Preclinical Evidence of Ketamine Antidepressant Effects

Modeling suicidal behaviors in animals represents a complex challenge, since suicide has not been identified in any animal species despite the thousands of studies performed. In particular, the challenge with animals is trying to model the process of ideation, will, and intention to suicide, which seem to be characteristics peculiar to humans [60]. However, the most recent preclinical approaches aim at modeling specific human endophenotypes that have been associated with the suicidal process. Some pathways associated to suicidal behaviors, such as hypothalamic-pituitary-adrenal axis dysfunction and stress, neurotransmission anomalies, neuroinflammatory and neuroendocrine changes, aggression, impulsivity, and cognitive biases may be well modeled in animals, thereby permitting testing of the efficacy of renowned (clozapine, lithium) and newly-discovered potential anti-suicidal treatments, such as ketamine, in eventually preventing suicide [61]. Here we describe the preclinical effects of ketamine on neurobiological pathways associated with depressive behaviors in animal models and potentially involved in human suicidal processes. 

Ketamine is a NMDA receptor antagonist that was approved in 1970 to be used as an anesthetic drug. It has a 2.5 h blood half-life and is metabolized to norketamine and dehydronorketamine via cytochrome P450 system.

Ketamine yields peculiar pharmacodynamic properties. It is a non-selective NMDA receptor antagonist acting at opened channels, but several studies have identified multiple receptors interactions of ketamine, such as with opioid sigma and mu receptors, serotonin 5HT3 receptors, muscarinic receptors, α7 nicotinic acetylcholine receptors, and cathecolamines transporters [62,63].

Usually, ketamine is administered as a 1:1 racemic (S,R) mixture, and (S)-ketamine (esketamine) has been demonstrated to display a higher affinity for phencyclidine NMDA binding sites as compared to the (R) isomer [64].

Based on the early evidence that the NMDA non-competitive antagonist MK-801 might display antidepressant properties by counteracting the stress-induced impairment in long-term potentiation (LTP) in the hippocampus [65], a huge amount of studies have been performed demonstrating the rapid antidepressant effects of ketamine in animal models. First studies reported that animals injected with ketamine showed lesser despair behaviors during a forced swing test (FST) as compared to controls [66]. Moreover, a direct correlation between reduction in immobility at the FST and increase in hippocampal brain-derived neurotrophic factor (BDNF) was noted in successive studies, thus posing the basis for the acknowledgement of the involvement of the tropomyosin receptor kinase B (TrkB) signaling pathway in the antidepressant mechanisms of action of ketamine [67].

Finally, the fact that a pretreatment with NBQX, an α-amino-3-hydroxy-5-methylisoxazole-4-propionic acid (AMPA) receptors inhibitor, might reduce the antidepressant effects exerted by ketamine during the FST pointed out the essential role of postsynaptic AMPA receptors activation besides presynaptic NMDA blockade in the molecular mechanisms underlying these actions [68]. Over the years, several studies have corroborated the abovementioned results, refining and perfecting our knowledge on the intra-neural underpinnings of ketamine antidepressant action. For example, in animal models of chronic unpredictable stress there is evidence that depression-like behaviors are accompanied by the hippocampal upregulation of inflammatory markers, such as of interleukin (IL)-1β, IL-6, tumor necrosis factor-α (TNF-α), indoleamine 2,3-dioxygenase (IDO), and kynurenine (KYN)/tryptophan (TRP) ratio, which can be attenuated by the administration of sub-anesthetic doses of ketamine [69].

Moreover, in animal models of maternal deprivation, ketamine has been demonstrated to reverse depressive-like behaviors after a single dose with sustained antidepressant effects, as well as to protect from oxidative stress-induced brain damage by increasing the activities of superoxide dismutase and catalase that were reduced in deprived animals [70]. Also, the same authors reported that the administration of ketamine to maternal-deprived rats may attenuate the induced increase in inflammatory cytokines (TNF-α and IL-1 in serum, and in IL-6 in serum and cerebrospinal fluid) [71]. In order to confirm the great potential of ketamine in the therapeutic approach to treatment-resistant depression (TRD), some studies have demonstrated that this compound may induce significant reduction in despair behaviors during FST also in repeated corticosterone-treated animals (which is a model of TRD) [72].

The vast majority of studies concerning antidepressant effects of ketamine have localized the principal sites of action of this compound in PFC and hippocampal brain areas. Indeed, ketamine microinfusion in infralimbic PFC has been demonstrated to be able to mimic the antidepressant effects of systemic ketamine infusion, and these effects may be also mimicked by optogenetic stimulation of the same brain area, being related to the increase in number and function of spine synapses of layer V pyramidal neurons [73]. Ketamine has also been reported to increase hippocampal-PFC connectivity (which has been demonstrated to be impaired in depression) in both humans and animals by inducing an hyperglutamatergic state measurable by means of an electroencephalographic gamma band power enhancement [74,75].

However, besides the peculiar modulation of oxidative stress enzymes and inflammatory markers, as well as the impact on multiple neurotransmissions, all the scientific literature on ketamine converges on the evidence that the antidepressant effects of this compound are essentially mediated by the AMPA-dependent activation in PFC and hippocampus of two crucial transduction pathways: the mammalian target of rapamycin (mTOR) and brain-derived neurotrophic factor (BDNF) signaling.

### 3.2. mTOR and BDNF: The Molecular Cores of Ketamine Antidepressant and Anti-Suicidal Action

mTOR is a highly conserved serine/threonine protein kinase, which has been described to modulate several important cell functions, such as metabolism, proliferation, death, and protein synthesis [76]. mTOR exerts its regulatory action on protein synthesis through the inhibitory phosphorylation of the repressor of mRNA translation, eukaryotic initiation factor 4E-binding protein (4E-BP1), as well as the activating phosphorylation of S6 kinase (S6K1) [76]. Rapamycin may inhibit mTOR functions by modulating its upstream regulators, the protein kinase B (PKB/Akt) and extracellular signal-related kinase (ERK), which in turn may modulate the tuberous sclerosis complexes (TSC1, TSC2), which are mTOR inhibitors [77]. The glucose synthase kinase-3 (GSK3) may inhibit mTOR functions through the activation of TSC1 and TSC2 [78]. The final regulation of protein translation by mTOR is exerted via the eukaryotic elongation factor 2 (eEF2), which may be inhibited by S6K1 [76]. mTOR may associate in two different complexes: mTOR complex 1 (mTORC1), which requires the regulatory-associated protein of mTOR (Raptor) and is modulated by rapamycin [79], and mTOR complex 2 (mTORC2), which is rapamycin-independent [80].

A lot of brain physiological processes have been described to be underlain by mTOR, such as neuronal excitability and survival, synaptic potentiation/depression, dendritic spine activity-induced maturation, and memory formation (see [80] for review). Therefore, it is not surprising that mTOR has been regarded as involved in multiple neuropsychiatric disorders, and particularly in depressive disorders, both in humans and in animal models [81,82].

Recent genomic studies have identified molecules belonging to mTOR signaling as valuable biomarkers for suicidal behaviors [83]. Moreover, mTOR has been specifically associated to suicide attempts in US veterans [84].

**Figure 1:**
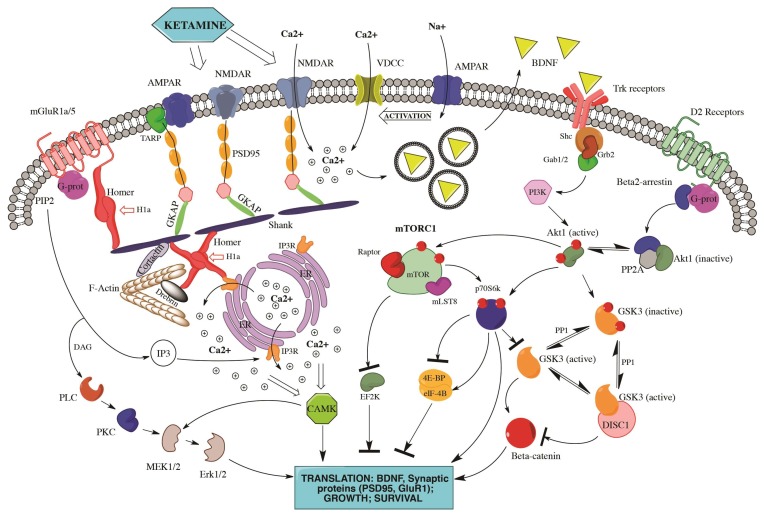
Complex molecular interactions amongst transductional pathways underlying ketamine antidepressant effects. NMDA receptors blocked by ketamine administration activates a complex downstream signaling, which comprises the entangled interaction among multiple transductional pathways, such as mTORC1-regulated transcription factors, PSD proteins, and calcium-modulated effectors. mTORC1 signaling is activated by AMPAR stimulation induced after NMDA blockade, which in turn may induce a Ca^2+^-mediated BDNF secretion and the subsequent Trk receptor stimulation, thus promoting translation of different factors deputed to the control of cell proliferation, growth and survival. PSD scaffolding proteins (Homer, Shank, PSD-95) connect multiple receptors, such as ionotropic and metabotropic glutamate receptors, as well linking these receptors to intracellular calcium stores. Through PSD proteins, glutamate signaling may intermingle at intracellular level with dopaminergic pathways, and activate GSK3, which may participate in the modulation of mTOR-regulated pathways, thereby affecting cell survival and differentiation. All these transductional pathways converge on appropriate nuclear targets via specific effectors (CaMK, MAPKsm ERK) in order to fine-modulate long-term activity-dependent neuronal rearrangements. NMDAR, *N*-methyl-d-aspartate glutamate receptor; AMPAR, α-amino-3-hydroxy-5-methyl-4-isoxazolepropionic acid glutamate receptor; mGluR1a/5, metabotropic glutamate receptor type 1a/5; VDCC, voltage-dependent calcium channel; BDNF, brain-derived neurotrophic factor; Trk receptors, tyrosine kinase receptors; TARP, transmembrane AMPA receptors regulating protein or stargazin; *PSD-95*, postsynaptic density protein 95kD; DISC1, disrupted in schizophrenia 1; GSK3, glycogen synthase kinase 3; GKAP, guanylate kinase associated protein; H1a, Homer1a immediate-early inducible protein; PIP2, phosphatydilinositol bisphosphate; DAG, diacylglycerol; IP3, inositol 1,4,5-trisphosphate; ER, endoplasmic reticulum; PLC, phospholipase C; PKC, protein kinase C; PP2A, protein phosphatase 2A; Akt1, RAC-α serine/threonine-protein kinase; CAMK, calcium-calmodulin regulated kinase; MAPKs, mitogen-activated protein kinases; Erk, extracellular signal-regulated kinase; MEK, MAPK/Erk kinase; Shc, Src homolog and collagen adaptor; Grb2, Growth factor receptor-bound protein 2; Gab1/2, Grb2-associated binder 1; PI3K, phosphoinositide 3 kinase; mTOR, mammalian target of rapamycin; Raptor, Regulatory-associated protein of mTOR; mLST8, mammalian lethal with SEC13 protein 8; p70S6K, ribosomal p70 S6 kinase; 4E-BP, 4E eukaryotic factor binding protein; eIF-4B, eukaryotic initiation factor 4B; EF2K, Eukaryotic elongation factor 2 kinase.

A pivotal study by Li et al. has demonstrated that the modulation of mTOR-dependent pathways is crucial for ketamine-induced antidepressant effects [85]. mTOR, indeed, represents an essential target of ketamine in the prefrontal cortex of animal models of depressive states, and the activation of mTOR by ketamine also provokes the increase in mRNA and protein levels of activity-regulated cytoskeletal-associated protein Arc, synapsin I, postsynaptic density protein 95 (PSD95), and GluR1 (an AMPA receptor subunit), all of which have been demonstrated to play core roles in synaptic plasticity [85], as shown in Figure 1. Moreover, a successive study by the same group reported that ketamine may not only attenuate anhedonic and anxiogenic behaviors induced by chronic unpredictable stress in animal models, but also revert the stress-induced decrease in expression levels of synaptic proteins (PSD95, synapsin I, GluR1), spine number, and frequency of excitatory postsynaptic currents [86].

The antidepressant effects of ketamine via mTOR activation have been demonstrated to require GluN2B-containing NMDA receptors in PFC, whose deletion may mimic and impede ketamine actions [87]. The application of AMPA receptor-blocking compounds may also inhibit ketamine’s effects, thereby emphasizing the essentiality of these receptors as mediators of its antidepressant action [68].

In order to exert its well-known rapid antidepressant effects, ketamine is compelled to activate two transductional processes that have been regarded as fundamental: the GSK3 and the BDNF pathways. GSK3 is a serine-threonine kinase, which has been crucially involved in depressive and bipolar disorders [88,89], as seen in Figure 1. Using knock-in transgenic animals with serine-to-alanine mutations to eliminate the inhibitory serines of GSK3, Beurel et al. demonstrated that when GSK3 cannot be inhibited ketamine is not able to display the usual antidepressant effects in the learned helplessness model of depression-like behavior [90]. Based on this study, other authors have reported that the add-on of lithium—a well-established GSK3 inhibitor—may potentiate the antidepressant effects of ketamine and increase its anti-oxidative action in PFC of animal models of depressive behaviors [91,92].

BDNF and mTOR pathways are tightly entangled and have been demonstrated to influence each other with a complicated feedback mechanism, as seen in Figure 1. The activation of mTOR, induced by the ketamine-mediated NMDA blockade, may de-suppress the protein translation machinery controlled by eEF2 (see above), thus promoting hippocampal BDNF expression, which seems crucial for ketamine’s antidepressant effects [82]. Indeed, in BDNF knock-out animals, ketamine is not able to produce antidepressant effects, thus demonstrating the crucial role of BDNF in fast-acting responses following ketamine administration [93]. Moreover, the release of BDNF from PFC neurons is essentially mediated by post-synaptic AMPA receptor stimulation, which is concurrently activated by ketamine NMDA blockade, and by the activation of L-type voltage-dependent calcium channels, which stimulate the activity-dependent exocytosis of BDNF [94].

The release of BDNF promotes a lot of synaptic plasticity mechanisms and dendritic spine modifications that have been proposed to underlie the long-term effects of ketamine administration [95], as well as of other antidepressants [96]. Moreover, BDNF has been reported to start a feedback re-stimulation of mTOR, through the activation of Tropomyosin receptor kinase B (TrkB) and its downstream effectors ERK and Akt, which in turn activate mTOR, as well as through the increase in synthesis and trafficking of AMPA receptors, thus increasing their signaling and AMPA-mediated mTOR stimulation [97], as shown in Figure 1.

The role of BDNF in ketamine antidepressant action is confirmed by studies demonstrating that ketamine efficacy is reduced in transgenic animals carrying the Val66Met BDNF mutation, which impairs ketamine-mediated synaptogenesis by altering trafficking of BDNF to dendrites [98]. Considering that a quarter-to-a third of the population has been estimated to carry the Val66Met BDNF mutation [99], this may account for the one third depressed patients who seem resistant to ketamine’s effects.

### 3.3. Comparing Anti-Suicidal Treatment Neurobiology: Ketamine vs. Classical Antidepressants, Lithium and Clozapine

Most of the data on the efficacy of classical treatments for suicidal behaviors rely on the efficacy of these treatments on the pathologies they have been prescribed for. In fact, diverse controversies exist on the efficacy of classical antidepressant treatments on both suicide ideation and attempts. Although a lot of studies confirm that generally both tricyclic and selective serotonin reuptake inhibiting (SSRI) antidepressants may lower the risk of suicide in treated patients [100,101,102,103], the FDA has given a warning on the use of antidepressants based on the evidence that these drugs may increase suicidality in children and adolescents [104]. These effects may depend upon the delayed action of antidepressant drugs, as well as on the possible emergence during the treatment of agitation, insomnia, mixed symptoms, and impulsivity, which are often perceived as unpleasant and may account for the increase in suicidal impulses [105]. From a neurobiological point of view, the incomplete efficacy of classical antidepressant treatment on suicidal behavior may be due to their selective modulation of serotonin neurotransmission, which, as previously described, has been only partially implicated in the pathophysiology of suicide processes. Indeed, although some polymorphisms in serotonergic neurotransmission-related genes (i.e., tryptophan hydroxylase, serotonin transporter) have been associated with temperamental traits in people prone to depression and suicide [106], no association has been found between polymorphisms in serotonin receptors and suicidal behaviors [107]. A recent metanalysis found no correlation between the 5-HTTLPR polymorphism and suicidal behavior, though a specific influence was found on the increased risk of violent suicide [108]. These results contribute to defining serotonin signaling as a part of the complex molecular machinery implicated in the pathophysiology of suicidal behaviors, thereby relegating classical antidepressants to partial anti-suicidal treatments, often ineffective or even counterproductive.

Differently from antidepressants, lithium treatment has been associated with a significant reduction in suicide risk and suicide attempts in patients with bipolar disorder or major depressive disorder, so much so that the American Psychiatric Association has put this statement in its Practice Guidelines for the assessment of patients with suicidal behavior from 2003 [109]. These effects have been confirmed in a recent metanalysis [110]. Interestingly, although for the most part of anti-suicidal treatments achieve their objective by treating depressive correlates, lithium has been described as reducing suicidal behaviors independently from mood improvement [111]. A large body of evidence suggests that the anti-suicidal effects of lithium may rely on its action on multiple levels of the suicidal process. Preclinical studies demonstrated that lithium may impact peculiar endophenotypes associated with suicidal behaviors, such as impulsivity, an action that is not shared with other mood stabilizers [112,113]. Differently from ketamine, whose effects may be measured in terms of hours or days after administration, lithium efficacy on suicidal behaviors arises in long-term treatment. However, a large amount of studies report that lithium anti-suicidal effects rely on the modulation of neurobiological pathways which are also impacted substantially by ketamine. Indeed, a crucial role in lithium transductional effects is exerted by GSK3 [114]. Through GSK3 activity regulation, lithium treatment may impact inflammatory cascades, which are mys-activated by HPA axis dysregulation in mood disorders, and have been associated with suicide-related impulsivity/aggression endophenotype [115]; moreover, as previously mentioned with regard to ketamine, GSK3 modulation is essential for oxidative stress regulation as well as BDNF secretion, both implicated in depressive disorders and suicidal behaviors [116]. GSK3 has been regarded also to act as a molecular crossroad in serotonin transductional pathways, being dysregulated in behavioral abnormalities induced in animal models of serotonin deficiency [117], thus representing a further possible target of lithium action. Last, but not least, the modulation of GSK3 by lithium has been regarded as a core target in the reinstatement of circadian rhythmicity disruption via the regulation of CLOCK genes, whose dysfunction has been strongly implicated in suicidal behaviors in patients suffering from major affective disorders [118].

Clozapine has been approved for the treatment of suicidal ideation in patients suffering from schizophrenia. Although there is a large evidence of these effects, to date the mechanisms of anti-suicidal properties of clozapine are still elusive. Some studies have suggested that clozapine efficacy on suicidal behaviors in schizophrenia patients may parallel its efficacy on treatment-refractory forms of the disorder, probably relying on the nonselective multireceptorial modulation exerted by this drug [119]. In preclinical studies, clozapine, similarly to lithium, has been demonstrated to prevent aggressive behaviors in animal models of depressive states, which represent an endophenotype of suicidal behavior in humans [120]. Clozapine appears efficacious also in controlling pro-suicidal traits in two-hit animal models of schizophrenia showing aggressiveness, impulsivity, hopelessness, and anxiety [121]. Moreover, the well-known modulation by clozapine of 5HT1a receptors, which leads to an increase in PFC dopamine release, has been involved in the clinical efficacy of this drug on both negative and cognitive symptoms of schizophrenia, whose treatment-refractoriness has been associated in turn to the propension to suicidal ideation in patients [122]. As in the case of ketamine and lithium, clozapine’s anti-suicidal effects seem to depend upon the modulation of multiple, and in large part unknown, transduction pathways. In order to confirm these theories, a recent study demonstrated that the beneficial effects of clozapine in reverting pro-suicidal like endophenotypes in two-hit animal models of schizophrenia may be impaired by the administration of the antiandrogen finasteride, thus suggesting a complex interaction between clozapine and neurosteroid signaling in the regulation of suicidal behavior [123].

## 4. Eradicating the Roots of Suicide: Clinical Randomized Studies of Anti-Suicidal Effects of Ketamine

For the purpose of this review only randomized clinical trials (RCT) were evaluated to investigate the efficacy of i.v. ketamine or intranasal esketamine on suicidal ideation. The efficacy of ketamine and esketamine has been evaluated in several randomized trials (see Table 1).

Since early studies, ketamine infusion (0.5 mg/kg) mixed with other anesthetic drugs (propofol, fentanyl) used to induce anesthesia in depressed patients undergoing orthopedic surgery was demonstrated to improve both depression and postoperative pain already one day after surgery [124]. The incredible speed of antidepressant action by ketamine has been repeatedly reported in successive RCTs, starting from Zarate et al. [125], who demonstrated that the first effects on depressive symptoms and suicidal thoughts may be seen only 40 min after ketamine injection (0.5 mg/kg 40 min i.v. infusion) in bipolar patients, and persist stably for three days, then gradually decreasing in the next two weeks, with only few patients maintaining results at this time point. Dissociative symptoms have been seen in the most part of patients, though not reaching impairing severity.

Similar results have been replicated in several RCTs, as shown in Table 1, demonstrating that ketamine infusion has rapid antidepressant and anti-suicidal effects starting soon after the administration, and lasting for a few (3–14) days with scarce side effects. This formidable action may be observed in all major affective disorders, and it seems independent from the concomitant medications administered (especially antidepressants or mood stabilizers).

Given the controversial literature reports on the major antidepressant effects of the administration of single isomers of ketamine rather than the racemic mixture [126], recent studies have focused on the possible administration of S-ketamine (esketamine, which is considered the most active and less damaging isomer) by intranasal administration. The administration of intranasal esketamine (84 mg) has been demonstrated to induce soon antidepressant effects (within 4 h), rapidly diminishing (up to a week) with scarce side effects (mostly dissociation, dizziness, and headache), and a peculiar action on suicidal thoughts, which rapidly decrease within 4 h from administration. However, these effects do not persist significantly after 24 h [127].

Overall, all reviewed trials demonstrated an efficacy of ketamine [125,128,129,130,131,132,133,134,135] and esketamine [127] in reducing suicidal ideation in patients with MDD, bipolar depression, cancer or other conditions. These results were also confirmed by a meta-analysis (based on 10 earlier open and randomized trials) that found that a single ketamine infusion rapidly reduced the severity of suicidal thinking, within 24 h in more than half the patients, and with benefits observed for up to 1 week. However, our review, even if not systematic, but narrative, included most recent trials and corroborated this observation.

The most common adverse effects (AEs) of ketamine were nausea, transient elevations in blood pressure (with intranasal formulation), drowsiness, dizziness, poor coordination, blurred vision, and feelings of strangeness or unreality, but these were not considered to be problematic for the subjects [125,127,128,129,130,131,132,133,134,135]. However, dissociative and psychotomimetic AEs occurred more commonly with ketamine, but were short-lasting and clinically not significant [127,128,129].

However, despite the potential benefits of ketamine as a “crisis” treatment for subjects with high suicide risk [136,137], there are still several concerns on its use as pointed out by Nemeroff [138] in a recent Editorial published on the American Journal of Psychiatry.

Obviously, the major concern is that ketamine is a substantial drug of abuse worldwide. This may be somewhat problematic, even if the employed dosages in randomized trials were very low, as it may promote abuse in predisposed subjects as reported after the conclusion of one trial [128] and other observations [138,139]. Furthermore, Nemeroff [138] underlines that many ketamine clinics that are operating in the USA do not follow the minimal recommendations of a recent American Psychiatric Association consensus report [140] and may result in the off-label prescribing of ketamine for patients with psychiatric disorders without a strong recommendation for its use [141].

Moreover, though there is robust evidence that ketamine may offer significant short-term benefits to many individuals suffering from possibly fatal mood disorders due to high suicide risk, this treatment has not yet undergone the test of multiple large-scale trials to determine the durability and safety of long-term treatment. Only Grunebaum et al. [128] found that a significant anti-suicidal effect was maintained for up to 6 weeks when combined with optimized pharmacotherapy, but there is a lack of studies that evaluated the ketamine effect on suicidal ideation after 6 weeks.

## 5. Eradicating the Roots of Suicide with Ketamine Treatment: Conclusions

Overall, the mechanism of action of ketamine in the treatment of suicidal patients involves several pathways but, first of all, the glutamatergic system seems to play a pivotal role [142]. In fact, it has been suggested that a glutamate neurotransmission dysregulation may be the basis of suicidal cognitive biases, explaining the benefits of ketamine treatment [53,143]. Ketamine is a non-selective NMDA receptor antagonist acting at opened channels, but several studies have identified multiple receptor interactions of ketamine, such as with opioid sigma and mu receptors, serotonin 5HT3 receptors, muscarinic receptors, α7 nicotinic acetylcholine receptors, and cathecolamines transporters, localizing the principal sites of action of this compound in PFC and hippocampal brain areas [144]. Therefore all these ketamine actions on neurotransmitters and selected brain areas may further contribute to its antidepressant and anti-suicidal properties [145].

Moreover, to explain its well-known rapid antidepressant and anti-suicidal effects, ketamine is compelled to activate two transductional processes that have been regarded as fundamental: the GSK3 and the BDNF pathways [146,147]. Also, ketamine action on mTOR-dependent pathways may further contribute to its rapid effects [148,149].

Concerning clinical trials, the results of this narrative review demonstrated a remarkable and fast efficacy of ketamine and esketamine (within 24 h in more than half of the patients, and with benefits observed for up to 1 week) in reducing suicidal ideation in patients with MDD, bipolar depression, cancer or other conditions. The most common adverse effects (AEs) of ketamine weren’t considered to be problematic in clinical randomized trials even if dissociative and psychotomimetic AEs occurred more commonly with ketamine than comparators (midazolam or placebo).

However, despite the potential benefits of ketamine as a “crisis” treatment of a subjects with high suicide risk, there are still several concerns on its use and the main may be the potential abuse of this compound and the lacking of multiple large-scale trials to determine the durability and safety of long-term ketamine treatment [136,141].

## Figures and Tables

**Table 1:** Randomized clinical trial of ketamine and esketamine on suicide ideation and risk.

Authors	Year	Study Sample	Study Design	Comparator	Number of Subject	Study Aims	Ketamine Dose	Main Findings
Canuso et al.	2018	Subjects with MDD and suicidal ideation	Randomized, placebo-controlled trial	Intranasal Placebo	68	To compared the efficacy of standard-of-care treatment plus intranasal esketamine or placebo for rapid reduction of symptoms of MDD, including suicidality	Intranasal esketamine (84 mg) twice weekly for 4 weeks	Intranasal esketamine compared with placebo, given in addition to comprehensive standard-of-care treatment, may result in significantly rapid improvement in depressive symptoms, including suicidal ideation
Grunebaum et al.	2018	Tested the acute effect of adjunctive subanesthetic intravenous ketamine on clinically significant suicidal ideation in patients with MDD	Randomized, controlled trial	Midazolam	80	To test the acute effect of adjunctive subanesthetic intravenous ketamine on clinically significant suicidal ideation in MDD	0.5 mg/kg infused over 40 min	Adjunctive ketamine demonstrated a greater reduction in clinically significant suicidal ideation in depressed patients within 24 h compared with midazolam, partially independently of antidepressant effect
Grunebaum et al.	2017	Subjects with bipolar depression and suicidal ideation	Pilot, randomized, controlled trial	Midazolam	16	To evaluate feasibility and effects of a sub-anesthetic infusion dose of ketamine versus midazolam on suicidal ideation in bipolar depression	0.5 mg/kg infused over 40 min	Suicidal thoughts were lower after ketamine than after midazolam at a trend level of significance
Fan et al.	2017	Cancer patients with suicidal ideation	Randomized, controlled trial	Midazolam	37	To evaluate the rapid antidepressant effects of single dose ketamine on suicidal ideation and overall depression level in patients with newly-diagnosed cancer	0.5 mg/kg infused over 40 min	Antidepressant and anti-suicidal effects of ketamine were significantly seen as soon as 1 day following administration and typically lasted for at least 3 days
Burger et al.	2016	Individuals presenting with suicidal thinking in a military setting	Randomized, placebo-controlled trial	Normal saline (placebo)	10	To evaluate the potential benefits of ketamine vs. placebo administered to individuals presenting with suicidal thinking in a military setting	0.5 mg/kg infused over 40 min	Two of three who received ketamine experienced dramatic decreases in suicidality and hopelessness within 40 min
Hu et al.	2016	Thirty outpatients with severe MDD	Randomized, placebo-controlled 4-week study combined with escitalopram 10 mg	Normal saline (placebo)	30	To evaluate the antidepressant and anti-suicidal effects of single i.v. ketamine combined with escitalopram initiation in MDD	0.5 mg/kg infused over 40 min	Single-dose i.v. ketamine showed an attenuation of the antidepressant effects by the end of week 1. Suicidal ideation improved significantly, which was sustained for up to 10 days
Murrough et al.	2016	Subjects with mood and anxiety disorders spectrum	Randomized, controlled trial	Midazolam	24	To evaluate acute effect of i.v. ketamine on clinically significant suicidal ideation in patients with mood and anxiety disorders spectrum	0.5 mg/kg infused over 40 min	A single adjunctive ketamine infusion was associated with a clinically significant reduction in suicidal ideation at 48 h that was greater than with the midazolam control infusion
Price et al.	2014	Symptomatic patients with treatment-resistant MDD	Randomized, controlled trial	Midazolam	57	To evaluate acute effect of i.v. ketamine vs midazolam on explicit and implicit suicidal cognition	0.5 mg/kg infused over 40 min	i.v. ketamine showed rapid reductions in suicidal cognition over and above midazolam
Zarate et al.	2012	Subjects with DSM-IV bipolar I or II depression maintained on therapeutic levels of lithium or valproate	Double-blind, randomized, crossover, placebo-controlled study	Normal saline (placebo)	15	To evaluate acute effect of i.v. ketamine on clinically significant suicidal ideation in patients with bipolar depression	0.5 mg/kg infused over 40 min	Within 40 min, depressive symptoms, as well as suicidal ideation, significantly improved in subjects receiving ketamine compared with placebo and this improvement remained significant through day 3
Kudoh et al.	2002	Depressed patients who had undergone orthopedic surgery	Randomized study. Patients in Group A were induced with propofol, fentanyl, and ketamine and patients in Group B were induced with propofol and fentanyl	Propofol, fentanyl	95	To evaluate acute effect of i.v. ketamine on clinically significant suicidal ideation in depressed patients who had undergone orthopedic surgery	0.5 mg/kg combined with propofol and fentanyl	Depressed mood, suicidal tendencies, somatic anxiety, and hypochondriasis significantly decreased in Group A as compared with Group B

## References

[1] World Health Organization (WHO) (2018). Suicide.

[2] World Health Organization (WHO) (2017). Suicide Data.

[3] Arsenault-Lapierre G., Kim C., Turecki G. (2004). Psychiatric diagnoses in 3275 suicides: A meta-analysis. BMC Psychiatry.

[4] Hegerl U. (2016). Prevention of suicidal behavior. Dialogues Clin. Neurosci..

[5] Pompili M., Gonda X., Serafini G., Innamorati M., Sher L., Amore M., Rihmer Z., Girardi P. (2013). Epidemiology of suicide in bipolar disorders: A systematic review of the literature. Bipolar Disord..

[6] Wasserman D., Wasserman C. (2009). Oxford Textbook of Suicidology and Suicide Prevention: A Global Perspective.

[7] Bertolote J.M., Fleischmann A., De Leo D., Wasserman D. (2004). Psychiatric Diagnoses and Suicide: Revisiting the Evidence. Crisis.

[8] Fountoulakis K.N., Kawohl W., Theodorakis P.N., Kerkhof A.J.F.M., Navickas A., Höschl C., Lecic-Tosevski D., Sorel E., Rancans E., Palova E. (2014). Relationship of suicide rates to economic variables in Europe: 2000–2011. Br. J. Psychiatry.

[9] De Berardis D., Martinotti G., Di Giannantonio M. (2018). Editorial: Understanding the Complex Phenomenon of Suicide: From Research to Clinical Practice. Front. Psychiatry.

[10] De Berardis D., Fornaro M., Orsolini L., Valchera A., Carano A., Vellante F., Perna G., Serafini G., Gonda X., Pompili M. (2017). Alexithymia and Suicide Risk in Psychiatric Disorders: A Mini-Review. Front. Psychiatry.

[11] Pompili M., Innamorati M., Vichi M., Masocco M., Vanacore N., Lester D., Serafini G., Dominici G., Girardi P., De Leo D. (2010). Suicide prevention among youths. Systematic review of available evidence-based interventions and implications for Italy. Minerva Pediatr..

[12] Serafini G., Muzio C., Piccinini G., Flouri E., Ferrigno G., Pompili M., Girardi P., Amore M. (2015). Life adversities and suicidal behavior in young individuals: A systematic review. Eur. Child Adolesc. Psychiatry.

[13] Orsolini L., Valchera A., Vecchiotti R., Tomasetti C., Iasevoli F., Fornaro M., De Berardis D., Perna G., Pompili M., Bellantuono C. (2016). Suicide during Perinatal Period: Epidemiology, Risk Factors, and Clinical Correlates. Front. Psychiatry.

[14] Large M., Kaneson M., Myles N., Myles H., Gunaratne P., Ryan C. (2016). Meta-Analysis of Longitudinal Cohort Studies of Suicide Risk Assessment among Psychiatric Patients: Heterogeneity in Results and Lack of Improvement over Time. PLoS ONE.

[15] Schrijvers D.L., Bollen J., Sabbe B.G.C. (2012). The gender paradox in suicidal behavior and its impact on the suicidal process. J. Affect. Disord..

[16] Zalsman G., Hawton K., Wasserman D., van Heeringen K., Arensman E., Sarchiapone M., Carli V., Hoschl C., Barzilay R., Balazs J. (2016). Suicide prevention strategies revisited: 10-year systematic review. Lancet Psychiatry.

[17] Madsen T., Karstoft K.-I., Secher R.G., Austin S.F., Nordentoft M. (2016). Trajectories of suicidal ideation in patients with first-episode psychosis: Secondary analysis of data from the OPUS trial. Lancet Psychiatry.

[18] Kasckow J., Youk A., Anderson S.J., Dew M.A., Butters M.A., Marron M.M., Begley A.E., Szanto K., Dombrovski A.Y., Mulsant B.H. (2016). Trajectories of suicidal ideation in depressed older adults undergoing antidepressant treatment. J. Psychiatr. Res..

[19] Köhler-Forsberg O., Madsen T., Behrendt-Møller I., Sylvia L., Bowden C.L., Gao K., Bobo W.V., Trivedi M.H., Calabrese J.R., Thase M. (2017). Trajectories of suicidal ideation over 6 months among 482 outpatients with bipolar disorder. J. Affect. Disord..

[20] Czyz E.K., King C.A. (2015). Longitudinal Trajectories of Suicidal Ideation and Subsequent Suicide Attempts Among Adolescent Inpatients. J. Clin. Child Adolesc. Psychol..

[21] Bostwick J.M., Pabbati C., Geske J.R., McKean A.J. (2016). Suicide Attempt as a Risk Factor for Completed Suicide: Even More Lethal Than We Knew. Am. J. Psychiatry.

[22] Turecki G., Ota V.K., Belangero S.I., Jackowski A., Kaufman J. (2014). Early life adversity, genomic plasticity, and psychopathology. Lancet Psychiatry.

[23] Oquendo M.A., Sullivan G.M., Sudol K., Baca-Garcia E., Stanley B.H., Sublette M.E., Mann J.J. (2014). Toward a Biosignature for Suicide. Am. J. Psychiatry.

[24] De Berardis D., Conti C.M., Serroni N., Moschetta F.S., Carano A., Salerno R.M., Cavuto M., Farina B., Alessandrini M., Janiri L. (2009). The role of cholesterol levels in mood disorders and suicide. J. Biol. Regul. Homeost Agents.

[25] Hariri A.R., Mattay V.S., Tessitore A., Kolachana B., Fera F., Goldman D., Egan M.F., Weinberger D.R. (2002). Serotonin transporter genetic variation and the response of the human amygdala. Science.

[26] Plomin R., Owen M.J., McGuffin P., Sabol S.Z., Greenberg B.D., Petri S., Benjamin J., Müller C.R., Hamer D.H., Murphy D.L. (1994). The genetic basis of complex human behaviors. Science.

[27] Bennett A., Lesch K., Heils A., Long J.C., Lorenz J.G., Shoaf S.E., Champoux M., Suomi S.J., Linnoila M.V., Higley J.D. (2007). Early experience and serotonin transporter gene variation interact to influence primate CNS function. Mol. Psychiatry.

[28] Caspi A., Sugden K., Moffitt T.E., Taylor A., Craig I.W., Harrington H., McClay J., Mill J., Martin J., Braithwaite A. (2003). Influence of Life Stress on Depression: Moderation by a Polymorphism in the 5-HTT Gene. Science.

[29] Sokolowski M., Ben-Efraim Y.J., Wasserman J., Wasserman D. (2013). Glutamatergic GRIN2B and polyaminergic ODC1 genes in suicide attempts: Associations and gene–environment interactions with childhood/adolescent physical assault. Mol. Psychiatry.

[30] Brodsky B.S. (2016). Early Childhood Environment and Genetic Interactions: The Diathesis for Suicidal Behavior. Curr. Psychiatry Rep..

[31] Jollant F., Lawrence N.L., Olié E., Guillaume S., Courtet P. (2011). The suicidal mind and brain: A review of neuropsychological and neuroimaging studies. World J. Boil. Psychiatry.

[32] Davis K.N., Tao R., Li C., Gao Y., Gondré-Lewis M.C., Lipska B.K., Shin J.H., Xie B., Ye T., Weinberger D.R. (2016). GAD2 Alternative Transcripts in the Human Prefrontal Cortex, and in Schizophrenia and Affective Disorders. PLoS ONE.

[33] Joiner T.E. Why People Die By Suicide 1 Why People Die By Suicide: Further Development and Tests of the Interpersonal-Psychological Theory of Suicidal Behavior. http://portal.idc.ac.il/en/symposium/hspsp/2011/documents/cjoiner11.pdf.

[34] Chopin E., Kerkhof A.J.F.M., Arensman E. (2004). Psychological dimensions of attempted suicide: Theories and data. Suicidal Behaviour: Theories and Research Findings.

[35] Wenzel A., Brown G.K., Beck A.T. (2009). Cognitive Therapy for Suicidal Patients: Scientific and Clinical Applications.

[36] Richard-Devantoy S., Turecki G., Jollant F. (2016). Neurobiology of Elderly Suicide. Arch. Suicide Res..

[37] Keilp J.G., Gorlyn M., Russell M., Oquendo M.A., Burke A.K., Harkavy-Friedman J., Mann J.J. (2013). Neuropsychological function and suicidal behavior: Attention control, memory and executive dysfunction in suicide attempt. Psychol. Med..

[38] McGirr A., Renaud J., Bureau A., Seguin M., Lesage A., Turecki G. (2008). Impulsive-aggressive behaviours and completed suicide across the life cycle: A predisposition for younger age of suicide. Psychol. Med..

[39] Dougherty D.M., Bjork J.M., Moeller F.G., Harper R.A., Marsh D.M., Mathias C.W., Swann A.C. (2003). Familial Transmission of Continuous Performance Test Behavior: Attentional and Impulsive Response Characteristics. J. Gen. Psychol..

[40] Malloy-Diniz L.F., Neves F.S., Abrantes S.S.C., Fuentes D., Corrêa H. (2009). Suicide behavior and neuropsychological assessment of type I bipolar patients. J. Affect. Disord..

[41] Wang Y.-Y., Jiang N.-Z., Cheung E.F.C., Sun H.-W., Chan R.C.K. (2015). Role of depression severity and impulsivity in the relationship between hopelessness and suicidal ideation in patients with major depressive disorder. J. Affect. Disord..

[42] Soloff P., White R., Diwadkar V.A. (2014). Impulsivity, aggression and brain structure in high and low lethality suicide attempters with borderline personality disorder. Psychiatry Res. Neuroimaging.

[43] Millan M.J., Agid Y., Brüne M., Bullmore E.T., Carter C.S., Clayton N.S., Connor R., Davis S., Deakin B., DeRubeis R.J. (2012). Cognitive dysfunction in psychiatric disorders: Characteristics, causes and the quest for improved therapy. Nat. Rev. Drug Discov..

[44] Anand A., Li Y., Wang Y., Wu J., Gao S., Bukhari L., Mathews V.P., Kalnin A., Lowe M.J. (2005). Activity and Connectivity of Brain Mood Regulating Circuit in Depression: A Functional Magnetic Resonance Study. Boil. Psychiatry.

[45] Koenigs M., Grafman J. (2009). The functional neuroanatomy of depression: Distinct roles for ventromedial and dorsolateral prefrontal cortex. Behav. Brain Res..

[46] Hasler G., van der Veen J.W., Tumonis T., Meyers N., Shen J., Drevets W.C. (2007). Reduced Prefrontal Glutamate/Glutamine and γ-Aminobutyric Acid Levels in Major Depression Determined Using Proton Magnetic Resonance Spectroscopy. Arch. Gen. Psychiatry.

[47] Feyissa A.M., Chandran A., Stockmeier C.A., Karolewicz B. (2009). Reduced levels of NR2A and NR2B subunits of NMDA receptor and PSD-95 in the prefrontal cortex in major depression. Prog. Neuro-Psychopharmacol. Boil. Psychiatry.

[48] Moghaddam B. (1993). Stress Preferentially Increases Extraneuronal Levels of Excitatory Amino Acids in the Prefrontal Cortex: Comparison to Hippocampus and Basal Ganglia. J. Neurochem..

[49] Yuen E.Y., Liu W., Karatsoreos I.N., Feng J., McEwen B.S., Yan Z. (2009). Acute stress enhances glutamatergic transmission in prefrontal cortex and facilitates working memory. Proc. Natl. Acad. Sci. USA.

[50] Dalton G.L., Ma L.M., Phillips A.G., Floresco S.B. (2011). Blockade of NMDA GluN2B receptors selectively impairs behavioral flexibility but not initial discrimination learning. Psychopharmacology.

[51] Yuen E.Y., Wei J., Liu W., Zhong P., Li X., Yan Z. (2012). Repeated Stress Causes Cognitive Impairment by Suppressing Glutamate Receptor Expression and Function in Prefrontal Cortex. Neuron.

[52] Jett J.D., Bulin S.E., Hatherall L.C., McCartney C.M., Morilak D.A. (2017). Deficits in cognitive flexibility induced by chronic unpredictable stress are associated with impaired glutamate neurotransmission in the rat medial prefrontal cortex. Neuroscience.

[53] Bernstein H.-G., Tausch A., Wagner R., Steiner J., Seeleke P., Walter M., Dobrowolny H., Bogerts B. (2013). Disruption of glutamate-glutamine-GABA cycle significantly impacts on suicidal behaviour: Survey of the literature and own findings on glutamine synthetase. CNS Neurol. Disord. Drug Targets.

[54] Gos T., Günther K., Bielau H., Dobrowolny H., Mawrin C., Trübner K., Brisch R., Steiner J., Bernstein H.-G., Jankowski Z. (2009). Suicide and depression in the quantitative analysis of glutamic acid decarboxylase-Immunoreactive neuropil. J. Affect. Disord..

[55] Naaijen J., Lythgoe D.J., Zwiers M.P., Hartman C.A., Hoekstra P.J., Buitelaar J.K., Aarts E. (2018). Anterior cingulate cortex glutamate and its association with striatal functioning during cognitive control. Eur. Neuropsychopharmacol..

[56] Khokhar J.Y., Henricks A.M., Sullivan E.D.K., Green A.I. (2018). Unique Effects of Clozapine: A Pharmacological Perspective. Adv. Pharmacol..

[57] Krakowski M.I., Czobor P. (2014). Depression and Impulsivity as Pathways to Violence: Implications for Antiaggressive Treatment. Schizophr. Bull..

[58] Serafini G., Adavastro G., Canepa G., Capobianco L., Conigliaro C., Pittaluga F., Murri M.B., Valchera A., De Berardis D., Pompili M. (2017). Abnormalities in Kynurenine Pathway Metabolism in Treatment-Resistant Depression and Suicidality: A Systematic Review. CNS Neurol. Disord. Drug Targets.

[59] Javitt D.C., Duncan L., Balla A., Sershen H. (2005). Inhibition of System A-mediated glycine transport in cortical synaptosomes by therapeutic concentrations of clozapine: Implications for mechanisms of action. Mol. Psychiatry.

[60] Preti A. (2011). Animal model and neurobiology of suicide. Prog. Neuropsychopharmacol. Biol. Psychiatry.

[61] Gould T.D., Georgiou P., Brenner L.A., Brundin L., Can A., Courtet P., Donaldson Z.R., Dwivedi Y., Guillaume S., Gottesman I.I. (2017). Animal models to improve our understanding and treatment of suicidal behavior. Transl. Psychiatry.

[62] Smith D.J., Azzaro A.J., Zaldivar S.B., Palmer S., Lee H.S. (1981). Properties of the optical isomers and metabolites of ketamine on the high affinity transport and catabolism of monoamines. Neuropharmacology.

[63] Smith D.J., Bouchal R.L., deSanctis C.A., Monroe P.J., Amedro J.B., Perrotti J.M., Crisp T. (1987). Properties of the interaction between ketamine and opiate binding sites in vivo and in vitro. Neuropharmacology.

[64] Vollenweider F.X., Leenders K.L., Oye I., Hell D., Angst J. (1997). Differential psychopathology and patterns of cerebral glucose utilisation produced by (S)- and (R)-ketamine in healthy volunteers using positron emission tomography (PET). Eur. Neuropsychopharmacol. J. Eur. Coll. Neuropsychopharmacol..

[65] Trullas R., Skolnick P. (1990). Functional antagonists at the NMDA receptor complex exhibit antidepressant actions. Eur. J. Pharmacol..

[66] Yilmaz A., Schulz D., Aksoy A., Canbeyli R. (2002). Prolonged effect of an anesthetic dose of ketamine on behavioral despair. Pharmacol. Biochem. Behav..

[67] Garcia L.S.B., Comim C.M., Valvassori S.S., Réus G.Z., Barbosa L.M., Andreazza A.C., Stertz L., Fries G.R., Gavioli E.C., Kapczinski F. (2008). Acute administration of ketamine induces antidepressant-like effects in the forced swimming test and increases BDNF levels in the rat hippocampus. Prog. Neuro-Psychopharmacol. Boil. Psychiatry.

[68] Maeng S., Zarate C.A., Du J., Schloesser R.J., McCammon J., Chen G., Manji H.K. (2008). Cellular Mechanisms Underlying the Antidepressant Effects of Ketamine: Role of α-Amino-3-Hydroxy-5-Methylisoxazole-4-Propionic Acid Receptors. Boil. Psychiatry.

[69] Wang N., Yu H.-Y., Shen X.-F., Gao Z.-Q., Yang C., Yang J.-J., Zhang G.-F. (2015). The rapid antidepressant effect of ketamine in rats is associated with down-regulation of pro-inflammatory cytokines in the hippocampus. Upsala J. Med. Sci..

[70] Réus G.Z., Carlessi A.S., Titus S.E., Abelaira H.M., Ignácio Z.M., da Luz J.R., Matias B.I., Bruchchen L., Florentino D., Vieira A. (2015). A single dose of S-ketamine induces long-term antidepressant effects and decreases oxidative stress in adulthood rats following maternal deprivation. Dev. Neurobiol..

[71] Réus G.Z., Nacif M.P., Abelaira H.M., Tomaz D.B., dos Santos M.A.B., Carlessi A.S., da Luz J.R., Gonçalves R.C., Vuolo F., Dal-Pizzol F. (2015). Ketamine ameliorates depressive-like behaviors and immune alterations in adult rats following maternal deprivation. Neurosci. Lett..

[72] Koike H., Iijima M., Chaki S. (2013). Effects of ketamine and LY341495 on the depressive-like behavior of repeated corticosterone-injected rats. Pharmacol. Biochem. Behav..

[73] Fuchikami M., Thomas A., Liu R., Wohleb E.S., Land B.B., DiLeone R.J., Aghajanian G.K., Duman R.S. (2015). Optogenetic stimulation of infralimbic PFC reproduces ketamine’s rapid and sustained antidepressant actions. Proc. Natl. Acad. Sci. USA.

[74] Grimm O., Gass N., Weber-Fahr W., Sartorius A., Schenker E., Spedding M., Risterucci C., Schweiger J.I., Böhringer A., Zang Z. (2015). Acute ketamine challenge increases resting state prefrontal-hippocampal connectivity in both humans and rats. Psychopharmacology.

[75] Gass N., Schwarz A.J., Sartorius A., Schenker E., Risterucci C., Spedding M., Zheng L., Meyer-Lindenberg A., Weber-Fahr W. (2014). Sub-Anesthetic Ketamine Modulates Intrinsic BOLD Connectivity Within the Hippocampal-Prefrontal Circuit in the Rat. Neuropsychopharmacology.

[76] Hay N., Sonenberg N. (2004). Upstream and downstream of mTOR. Genes Dev..

[77] Fingar D.C., Blenis J. (2004). Target of rapamycin (TOR): An integrator of nutrient and growth factor signals and coordinator of cell growth and cell cycle progression. Oncogene.

[78] Inoki K., Ouyang H., Zhu T., Lindvall C., Wang Y., Zhang X., Yang Q., Bennett C., Harada Y., Stankunas K. (2006). TSC2 Integrates Wnt and Energy Signals via a Coordinated Phosphorylation by AMPK and GSK3 to Regulate Cell Growth. Cell.

[79] Kim D.-H., Sarbassov D.D., Ali S.M., King J.E., Latek R.R., Erdjument-Bromage H., Tempst P., Sabatini D.M. (2002). mTOR Interacts with Raptor to Form a Nutrient-Sensitive Complex that Signals to the Cell Growth Machinery. Cell.

[80] Sarbassov D.D., Ali S.M., Sabatini D.M. (2005). Growing roles for the mTOR pathway. Curr. Opin. Cell Boil..

[81] Lu Y., Wang C., Xue Z., Li C., Zhang J., Zhao X., Liu A., Wang Q., Zhou W. (2015). PI3K/AKT/mTOR Signaling-Mediated Neuropeptide VGF in the Hippocampus of Mice Is Involved in the Rapid Onset Antidepressant-Like Effects of GLYX-13. Int. J. Neuropsychopharmacol..

[82] Bockaert J., Marin P. (2015). mTOR in Brain Physiology and Pathologies. Physiol. Rev..

[83] Niculescu A.B., Levey D.F., Phalen P.L., Le-Niculescu H., Dainton H.D., Jain N., Belanger E., James A., George S., Weber H. (2015). Understanding and predicting suicidality using a combined genomic and clinical risk assessment approach. Mol. Psychiatry.

[84] Flory J.D., Donohue D., Muhie S., Yang R., Miller S.A., Hammamieh R., Ryberg K., Yehuda R. (2017). Gene expression associated with suicide attempts in US veterans. Transl. Psychiatry.

[85] Li N., Lee B., Liu R.-J., Banasr M., Dwyer J.M., Iwata M., Li X.-Y., Aghajanian G., Duman R.S. (2010). mTOR-dependent synapse formation underlies the rapid antidepressant effects of NMDA antagonists. Science.

[86] Li N., Liu R.-J., Dwyer J.M., Banasr M., Lee B., Son H., Li X.-Y., Aghajanian G., Duman R.S. (2011). Glutamate N-methyl-D-aspartate Receptor Antagonists Rapidly Reverse Behavioral and Synaptic Deficits Caused by Chronic Stress Exposure. Boil. Psychiatry.

[87] Miller O.H., Yang L., Wang C.-C., Hargroder E.A., Zhang Y., Delpire E., Hall B.J. (2014). GluN2B-containing NMDA receptors regulate depression-like behavior and are critical for the rapid antidepressant actions of ketamine. eLife.

[88] liu Z., Guo H., Cao X., Cheng C., Yang C., Xu C., Zhang A., Sun N., Li X., Zhang K. (2014). A combined study of GSK3β polymorphisms and brain network topological metrics in major depressive disorder. Psychiatry Res. Neuroimaging.

[89] Tang H., Shen N., Jin H., Liu D., Miao X., Zhu L.-Q. (2013). GSK-3β Polymorphism Discriminates Bipolar Disorder and Schizophrenia: A Systematic Meta-Analysis. Mol. Neurobiol..

[90] Beurel E., Song L., Jope R.S. (2011). Inhibition of glycogen synthase kinase-3 is necessary for the rapid antidepressant effect of ketamine in mice. Mol. Psychiatry.

[91] Liu R.-J., Fuchikami M., Dwyer J.M., Lepack A.E., Duman R.S., Aghajanian G.K. (2013). GSK-3 inhibition potentiates the synaptogenic and antidepressant-like effects of subthreshold doses of ketamine. Neuropsychopharmacol. Off. Publ. Am. Coll. Neuropsychopharmacol..

[92] Chiu C.-T., Scheuing L., Liu G., Liao H.-M., Linares G.R., Lin D., Chuang D.-M. (2014). The mood stabilizer lithium potentiates the antidepressant-like effects and ameliorates oxidative stress induced by acute ketamine in a mouse model of stress. Int. J. Neuropsychopharmacol..

[93] Autry A.E., Adachi M., Nosyreva E., Na E.S., Los M.F., Cheng P.-F., Kavalali E.T., Monteggia L.M. (2011). NMDA receptor blockade at rest triggers rapid behavioural antidepressant responses. Nature.

[94] Lepack A.E., Fuchikami M., Dwyer J.M., Banasr M., Duman R.S. (2014). BDNF release is required for the behavioral actions of ketamine. Int. J. Neuropsychopharmacol..

[95] Kavalali E.T., Monteggia L.M. (2012). Synaptic Mechanisms Underlying Rapid Antidepressant Action of Ketamine. Am. J. Psychiatry.

[96] Monteggia L.M., Zarate C. (2015). Antidepressant actions of ketamine: From molecular mechanisms to clinical practice. Curr. Opin. Neurobiol..

[97] Ignácio Z.M., Réus G.Z., Arent C.O., Abelaira H.M., Pitcher M.R., Quevedo J. (2016). New perspectives on the involvement of mTOR in depression as well as in the action of antidepressant drugs. Br. J. Clin. Pharmacol..

[98] Liu R.-J., Lee F.S., Li X.-Y., Bambico F., Duman R.S., Aghajanian G.K. (2012). Brain-derived neurotrophic factor Val66Met allele impairs basal and ketamine-stimulated synaptogenesis in prefrontal cortex. Boil. Psychiatry.

[99] Petryshen T.L., Sabeti P.C., Aldinger K.A., Fry B., Fan J.B., Schaffner S.F., Waggoner S.G., Tahl A.R., Sklar P. (2010). Population genetic study of the brain-derived neurotrophic factor (BDNF) gene. Mol. Psychiatry.

[100] Filakovic P., Eric A.P. (2013). Pharmacotherapy of suicidal behaviour in major depression, schizophrenia and bipolar disorder. Coll. Antropol..

[101] Yerevanian B.I., Choi Y.M. (2013). Impact of psychotropic drugs on suicide and suicidal behaviors. Bipolar Disord..

[102] Yerevanian B.I., Koek R.J., Feusner J.D., Hwang S., Mintz J. (2004). Antidepressants and suicidal behaviour in unipolar depression. Acta Psychiatr. Scand..

[103] Barbui C., Esposito E., Cipriani A. (2009). Selective serotonin reuptake inhibitors and risk of suicide: A systematic review of observational studies. CMAJ.

[104] Umetsu R., Abe J., Ueda N., Kato Y., Matsui T., Nakayama Y., Kinosada Y., Nakamura M. (2015). Association between Selective Serotonin Reuptake Inhibitor Therapy and Suicidality: Analysis of U.S. Food and Drug Administration Adverse Event Reporting System Data. Biol. Pharm. Bull..

[105] Goldsmith L., Moncrieff J. (2011). The psychoactive effects of antidepressants and their association with suicidality. Curr. Drug Saf..

[106] Pawlak J., Dmitrzak-Weglarz M., Maciukiewicz M., Kapelski P., Czerski P., Leszczynska-Rodziewicz A., Zaremba D., Hauser J. (2017). Personality traits as an endophenotype in genetic studies on suicidality in bipolar disorder. Acta Neuropsychiatr..

[107] Hofer P., Schosser A., Calati R., Serretti A., Massat I., Kocabas N.A., Konstantinidis A., Mendlewicz J., Souery D., Zohar J. (2016). The impact of serotonin receptor 1A and 2A gene polymorphisms and interactions on suicide attempt and suicide risk in depressed patients with insufficient response to treatment--a European multicentre study. Int. Clin. Psychopharmacol..

[108] Fanelli G., Serretti A. (2018). The influence of the serotonin transporter gene 5-HTTLPR polymorphism on suicidal behaviors: A meta-analysis. Prog. Neuropsychopharmacol. Biol. Psychiatry.

[109] Jacob D., Baldessarini R., Conwell Y., Fawcett J., Horton L., Meltzer H., Pfeffer C., Simon R. (2003). Practice guideline for the assessment and treatment of patients with suicidal behaviors. Am. J. Psychiatry.

[110] Tondo L., Baldessarini R.J. (2018). Antisuicidal Effects in Mood Disorders: Are They Unique to Lithium?. Pharmacopsychiatry.

[111] Toffol E., Hatonen T., Tanskanen A., Lonnqvist J., Wahlbeck K., Joffe G., Tiihonen J., Haukka J., Partonen T. (2015). Lithium is associated with decrease in all-cause and suicide mortality in high-risk bipolar patients: A nationwide registry-based prospective cohort study. J. Affect. Disord..

[112] Halcomb M.E., Gould T.D., Grahame N.J. (2013). Lithium, but not valproate, reduces impulsive choice in the delay-discounting task in mice. Neuropsychopharmacology.

[113] Ohmura Y., Tsutsui-Kimura I., Kumamoto H., Minami M., Izumi T., Yamaguchi T., Yoshida T., Yoshioka M. (2012). Lithium, but not valproic acid or carbamazepine, suppresses impulsive-like action in rats. Psychopharmacology.

[114] Beurel E., Jope R.S. (2014). Inflammation and lithium: Clues to mechanisms contributing to suicide-linked traits. Transl. Psychiatry.

[115] Malhi G.S., Outhred T. (2016). Therapeutic Mechanisms of Lithium in Bipolar Disorder: Recent Advances and Current Understanding. CNS Drugs.

[116] Can A., Schulze T.G., Gould T.D. (2014). Molecular actions and clinical pharmacogenetics of lithium therapy. Pharmacol. Biochem. Behav..

[117] Beaulieu J.M., Zhang X., Rodriguiz R.M., Sotnikova T.D., Cools M.J., Wetsel W.C., Gainetdinov R.R., Caron M.G. (2008). Role of GSK3 beta in behavioral abnormalities induced by serotonin deficiency. Proc. Natl. Acad. Sci. USA.

[118] Pawlak J., Dmitrzak-Weglarz M., Maciukiewicz M., Wilkosc M., Leszczynska-Rodziewicz A., Zaremba D., Kapelski P., Hauser J. (2015). Suicidal behavior in the context of disrupted rhythmicity in bipolar disorder--data from an association study of suicide attempts with clock genes. Psychiatry Res..

[119] Kim D.H., Maneen M.J., Stahl S.M. (2009). Building a better antipsychotic: Receptor targets for the treatment of multiple symptom dimensions of schizophrenia. Neurotherapeutics.

[120] Yang C.R., Bai Y.Y., Ruan C.S., Zhou H.F., Liu D., Wang X.F., Shen L.J., Zheng H.Y., Zhou X.F. (2015). Enhanced aggressive behaviour in a mouse model of depression. Neurotox. Res..

[121] Deslauriers J., Belleville K., Beaudet N., Sarret P., Grignon S. (2016). A two-hit model of suicide-trait-related behaviors in the context of a schizophrenia-like phenotype: Distinct effects of lithium chloride and clozapine. Physiol. Behav..

[122] Celada P., Bortolozzi A., Artigas F. (2013). Serotonin 5-HT1A receptors as targets for agents to treat psychiatric disorders: Rationale and current status of research. CNS Drugs.

[123] Maurice-Gelinas C., Deslauriers J., Monpays C., Sarret P., Grignon S. (2018). The 5α-reductase inhibitor finasteride increases suicide-related aggressive behaviors and blocks clozapine-induced beneficial effects in an animal model of schizophrenia. Physiol. Behav..

[124] Kudoh A., Katagai H., Takazawa T. (2002). Anesthesia with ketamine, propofol, and fentanyl decreases the frequency of postoperative psychosis emergence and confusion in schizophrenic patients. J. Clin. Anesth..

[125] Zarate C.A., Brutsche N.E., Ibrahim L., Franco-Chaves J., Diazgranados N., Cravchik A., Selter J., Marquardt C.A., Liberty V., Luckenbaugh D.A. (2012). Replication of ketamine’s antidepressant efficacy in bipolar depression: A randomized controlled add-on trial. Biol. Psychiatry.

[126] Andrade C. (2017). Ketamine for Depression, 3: Does Chirality Matter?. J. Clin. Psychiatry.

[127] Canuso C.M., Singh J.B., Fedgchin M., Alphs L., Lane R., Lim P., Pinter C., Hough D., Sanacora G., Manji H. (2018). Efficacy and Safety of Intranasal Esketamine for the Rapid Reduction of Symptoms of Depression and Suicidality in Patients at Imminent Risk for Suicide: Results of a Double-Blind, Randomized, Placebo-Controlled Study. Am. J. Psychiatry.

[128] Grunebaum M.F., Galfalvy H.C., Choo T.H., Keilp J.G., Moitra V.K., Parris M.S., Marver J.E., Burke A.K., Milak M.S., Sublette M.E. (2018). Ketamine for Rapid Reduction of Suicidal Thoughts in Major Depression: A Midazolam-Controlled Randomized Clinical Trial. Am. J. Psychiatry.

[129] Grunebaum M.F., Ellis S.P., Keilp J.G., Moitra V.K., Cooper T.B., Marver J.E., Burke A.K., Milak M.S., Sublette M.E., Oquendo M.A. (2017). Ketamine versus midazolam in bipolar depression with suicidal thoughts: A pilot midazolam-controlled randomized clinical trial. Bipolar Disord..

[130] Fan W., Yang H., Sun Y., Zhang J., Li G., Zheng Y., Liu Y. (2017). Ketamine rapidly relieves acute suicidal ideation in cancer patients: A randomized controlled clinical trial. Oncotarget.

[131] Burger J., Capobianco M., Lovern R., Boche B., Ross E., Darracq M.A., McLay R. (2016). A Double-Blinded, Randomized, Placebo-Controlled Sub-Dissociative Dose Ketamine Pilot Study in the Treatment of Acute Depression and Suicidality in a Military Emergency Department Setting. Mil. Med..

[132] Murrough J.W., Soleimani L., DeWilde K.E., Collins K.A., Lapidus K.A., Iacoviello B.M., Lener M., Kautz M., Kim J., Stern J.B. (2015). Ketamine for rapid reduction of suicidal ideation: A randomized controlled trial. Psychol. Med..

[133] Price R.B., Iosifescu D.V., Murrough J.W., Chang L.C., Al Jurdi R.K., Iqbal S.Z., Soleimani L., Charney D.S., Foulkes A.L., Mathew S.J. (2014). Effects of ketamine on explicit and implicit suicidal cognition: A randomized controlled trial in treatment-resistant depression. Depress Anxiety.

[134] Kudoh A., Takahira Y., Katagai H., Takazawa T. (2002). Small-dose ketamine improves the postoperative state of depressed patients. Anesth. Analg..

[135] Hu Y.D., Xiang Y.T., Fang J.X., Zu S., Sha S., Shi H., Ungvari G.S., Correll C.U., Chiu H.F., Xue Y. (2016). Single i.v. ketamine augmentation of newly initiated escitalopram for major depression: Results from a randomized, placebo-controlled 4-week study. Psychol. Med..

[136] Andrade C. (2018). Ketamine for Depression, 6: Effects on Suicidal Ideation and Possible Use as Crisis Intervention in Patients at Suicide Risk. J. Clin. Psychiatry.

[137] Andrade C. (2017). Ketamine for Depression, 2: Diagnostic and Contextual Indications. J. Clin. Psychiatry.

[138] Nemeroff C.B. (2018). Ketamine: Quo Vadis?. Am. J. Psychiatry.

[139] Schak K.M., Vande Voort J.L., Johnson E.K., Kung S., Leung J.G., Rasmussen K.G., Palmer B.A., Frye M.A. (2016). Potential Risks of Poorly Monitored Ketamine Use in Depression Treatment. Am. J. Psychiatry.

[140] Sanacora G., Frye M.A., McDonald W., Mathew S.J., Turner M.S., Schatzberg A.F., Summergrad P., Nemeroff C.B. (2017). American Psychiatric Association (APA) Council of Research Task Force on Novel Biomarkers and Treatments. A Consensus Statement on the Use of Ketamine in the Treatment of Mood Disorders. JAMA Psychiatry.

[141] Sanacora G., Heimer H., Hartman D., Mathew S.J., Frye M., Nemeroff C., Robinson Beale R. (2017). Balancing the Promise and Risks of Ketamine Treatment for Mood Disorders. Neuropsychopharmacology.

[142] Tomasetti C., Iasevoli F., Buonaguro E.F., De Berardis D., Fornaro M., Fiengo A.L., Martinotti G., Orsolini L., Valchera A., Di Giannantonio M. (2017). Treating the Synapse in Major Psychiatric Disorders: The Role of Postsynaptic Density Network in Dopamine-Glutamate Interplay and Psychopharmacologic Drugs Molecular Actions. Int. J. Mol. Sci..

[143] Zhao J., Verwer R.W., van Wamelen D.J., Qi X.R., Gao S.F., Lucassen P.J., Swaab D.F. (2016). Prefrontal changes in the glutamate-glutamine cycle and neuronal/glial glutamate transporters in depression with and without suicide. J. Psychiatr. Res..

[144] Andrade C. (2017). Ketamine for Depression, 1: Clinical Summary of Issues Related to Efficacy, Adverse Effects, and Mechanism of Action. J. Clin. Psychiatry.

[145] Duman R.S. (2018). Ketamine and rapid-acting antidepressants: A new era in the battle against depression and suicide. F1000Res.

[146] Zarate C.A., Machado-Vieira R. (2016). GSK-3: A key regulatory target for ketamine’s rapid antidepressant effects mediated by enhanced AMPA to NMDA throughput. Bipolar Disord..

[147] Le Nedelec M., Glue P., Winter H., Goulton C., Broughton L., Medlicott N. (2018). Acute low-dose ketamine produces a rapid and robust increase in plasma BDNF without altering brain BDNF concentrations. Drug Deliv. Transl. Res..

[148] Abelaira H.M., Reus G.Z., Ignacio Z.M., Dos Santos M.A., de Moura A.B., Matos D., Demo J.P., da Silva J.B., Michels M., Abatti M. (2017). Effects of ketamine administration on mTOR and reticulum stress signaling pathways in the brain after the infusion of rapamycin into prefrontal cortex. J. Psychiatr. Res..

[149] Zhou W., Wang N., Yang C., Li X.M., Zhou Z.Q., Yang J.J. (2014). Ketamine-induced antidepressant effects are associated with AMPA receptors-mediated upregulation of mTOR and BDNF in rat hippocampus and prefrontal cortex. Eur. Psychiatry.

